# Selection and Validation of Reference Genes for Quantitative Real-Time PCR Normalization in *Athetis dissimilis* (Lepidoptera: Noctuidae) Under Different Conditions

**DOI:** 10.3389/fphys.2022.842195

**Published:** 2022-02-22

**Authors:** Jinrong Tang, Gemei Liang, Shaoqi Dong, Shuang Shan, Man Zhao, Xianru Guo

**Affiliations:** ^1^Henan International Laboratory for Green Pest Control/College of Plant Protection, Henan Agricultural University, Zhengzhou, China; ^2^State Key Laboratory for Biology of Plant Diseases and Insect Pests, Institute of Plant Protection, Chinese Academy of Agricultural Sciences, Beijing, China

**Keywords:** reference gene, *Athetis dissimilis*, quantitative RT-PCR, gene expression stability, biotic and abiotic conditions

## Abstract

Reference genes are the key to study gene expression patterns using quantitative real-time PCR (qRT-PCR). No studies on the reference genes of *Athetis dissimilis*, an important agricultural pest, have been reported. In order to determine the reference genes for qRT-PCR normalization in *A. dissimilis* under different conditions, 10 candidate genes [18S ribosomal protein (*18S*), 28S ribosomal protein (*28S*), arginine kinase (*AK*), elongation factor 1 alpha (*EF1-α*), glyceraldehyde-3-phosphate dehydrogenase (*GAPDH*), ribosomal protein L32 (*RPL32*), ribosomal protein L40 (*RPL40*), alpha-tubulin (*α-TUB*), beta-actin (*β-ACT*), and beta-tubulin (*β-TUB*)] of *A. dissimilis* were selected to evaluate their stability as reference genes under different biotic and abiotic conditions by using five tools, geNorm, NormFinder, BestKeeper, ΔCt, and RefFinder. Furthermore, *CSP1* and superoxide dismutase (*SOD*) were used as target genes to validate the candidate reference genes. The results showed that different reference genes were needed under different experimental conditions, among which, *EF-1α*, *RPL40*, and *18S* are most suitable reference genes for studying genes related development stages of *A. dissimilis*, *RPL40* and *α-TUB* for larval tissues, *α-TUB* and *28S* for adult tissues, *EF-1α* and *β-ACT* for insecticidal treatments, *β-ACT* and *28S* for temperature treatments, *EF-1α* and *β-ACT* for starvation treatments, *RPL40* and *18S* for dietary treatments, and *18S*, *28S*, and *α-TUB* for all the samples. These results provide suitable reference genes for studying gene expression in *A. dissimilis* under different experimental conditions, and also lay the foundation for further research into the function of related genes in *A. dissimilis*.

## Introduction

In molecular biological research, gene expression analyses provide the information concerning gene regulatory mechanisms and functions associated with different biological processes (Zhao et al., 2018). Although there are many methods for evaluating gene expression profiles, quantitative real-time PCR (qRT-PCR) has been the most commonly used tool over the past several decades owing to its convenience, rapidity, specificity, and high sensitivity (Bustin et al., 2005; Huggett et al., 2005; Derveaux et al., 2010). For qRT-PCR studies, it is essential to select an appropriate reference gene to normalize qRT-PCR data in order to minimize the influence of RNA quality, reverse transcription efficiency, and PCR reaction conditions on the data. Thus, it is important to evaluate the validity of the candidate reference gene before qRT-PCR tests (Radonic et al., 2004; Huggett et al., 2005; Fleige and Pfaffl, 2006; Guenin et al., 2009; Xie et al., 2021).

An ideal reference gene should have steady expression levels under all the experimental conditions (Brym et al., 2013; Janska et al., 2013). However, the expression profiles of the widely used reference genes are not always constant under different experimental conditions (Selvey et al., 2001; Glare et al., 2002; Radonic et al., 2004). Numerous studies have demonstrated that even the same reference gene exhibited different expression levels under different experimental conditions, such as organismal developmental stages (Sun et al., 2009; Nakamura et al., 2016), tissues (Huis et al., 2010), cells (Nelissen et al., 2010), and temperatures (Mahanty et al., 2017). Therefore, it is crucial to identify suitable reference genes and evaluate their expression stability in certain target species under specific experimental conditions before the qRT-PCR data normalization of target gene expression levels using the reference genes (Guo et al., 2016; Wan et al., 2017; Renard et al., 2018).

For some model organisms or important economic insects, suitable reference genes for qRT-PCR have been identified and validated under various biotic or abiotic conditions (Fu et al., 2013; Yang et al., 2014; Zhang et al., 2015), but no appropriate reference genes have been identified and validated in *Athetis dissimilis*, an important insect pest widely distributed across many Asian countries (Dong et al., 2016; Sun et al., 2016; Guo et al., 2017; Che et al., 2019; Liu et al., 2019). The larvae of *A. dissimilis* bore into the seedling roots of maize, wheat, soybean, peanut, and other crop plants, causing the plants wilting and even death. Since it was first reported in Shandong Province, China in 2012, the damage of *A. dissimilis* has spread to Hebei, Henan, Shanxi, and other provinces. The development, mating behavior, and reproduction behavior of *A. dissimilis*, as well as its resistance to pesticide, are all regulated by related genes, and suitable reference genes are crucial to verify the gene expression profiles in *A. dissimilis* under different abiotic and biotic conditions. In addition, analysis of gene expression patterns under different conditions can provide valuable information regarding gene function and contribute to identify the important genes that may participate in these physiological and biological processes in *A. dissimilis*. However, the selection of reference genes of *A. dissimilis* in previous studies were all based on experience (Dong et al., 2016; Liu et al., 2019), a comprehensive study on the suitability of reference genes for qRT-PCR data normalization under different conditions is lacking.

Here, to identify suitable reference genes for the molecular study of *A. dissimilis*, 10 commonly used housekeeping genes, 18S ribosomal protein (*18S*), 28S ribosomal protein (*28S*), arginine kinase (*AK*), elongation factor 1 alpha (*EF1-α*), glyceraldehyde-3-phosphate dehydrogenase (*GAPDH*), ribosomal protein L32 (*RPL32*), ribosomal protein L40 (*RPL40*), alpha-tubulin (*α-TUB*), beta-actin (*β-ACT*), and beta-tubulin (*β-TUB*), were selected as the candidate reference genes (Ma et al., 2016; Yan et al., 2016; Gao et al., 2017; Hu et al., 2018; Yin et al., 2020), and their expression stabilities were analyzed under different biotic and abiotic conditions. Finally, a ranking of the stable reference genes was recommended for the corresponding experimental conditions. This work will provide suitable normalization genes for future gene expression studies and functional genomics research on *A. dissimilis* and its related species.

## Materials and Methods

### Insects

The larvae of *A. dissimilis* were provided by the Cotton Pest Research Group, Institute of Plant Protection, Henan Academy of Agricultural Sciences, Zhengzhou, China, and then maintained in the laboratory at 25 ± 1°C and 70 ± 5% relative humidity under a 14-h day:10-h night cycle (Guo et al., 2017). Larvae were reared on an artificial diet, and adults were provided with a 10% (w/w) honey solution.

### Candidate Reference Gene Clones and qRT-PCR Primer Design

The *β-ACT*, *RPL32*, *RPL40*, *EF1-α*, *α-TUB*, *β-TUB*, *18S*, *28S*, and *AK* genes in *A. dissimilis* were cloned as described below using the primers listed in Supplementary Table S1, and the sequence of *GAPDH* (GenBank accession no. KT361883.1) was downloaded from NCBI. Total RNAs from fourth-instar larvae of *A. dissimilis* were extracted using TRIzol reagent (Invitrogen, Carlsbad, CA, United States) in accordance with the manufacturer’s protocol. The purity and integrity of the total RNA were evaluated using a NanoVue spectrophotometer (GE Healthcare, United States). Then, genomic DNA in the RNA samples was removed using DNase I (TaKaRa, Japan), and the first-strand cDNA was synthesized using a PrimeScript first Strand cDNA Synthesis Kit (TaKaRa) in accordance with the manufacturer’s protocols. The PCR amplification conditions for these genes included a pre-denaturing step at 94°C for 3 min, followed by 35 cycles of 94°C for 30 s, 52°C for 40 s, and 72°C for 2 min, followed by a final extension at 72°C for 10 min. Afterward, the PCR products were gel purified using a DNA gel extraction kit (Tiangen Biotech, China), sub-cloned into the pMD19-T vector (TaKaRa), and then transformed into *Escherichia coli* DH5α cells. The positive clones were confirmed by PCR and sequenced (Supplementary Figure S1). These sequences have been submitted to NCBI (Table 1).

**Table 1:** Primer sequences and details of the candidate genes used in quantitative real-time PCR (qRT-PCR).

Gene name	Accession no.	Sequence (5–3′)	Amplicon size (bp)	Primer efficiency (%)	Regression coefficient (*R*^2^)	Linear regression
*GAPDH*	KT361883.1	F:CAAGATGGCTTCCTCGTA	161	96.74	0.9996	*y* = 3.4025*x* + 14.823
		R:GCACCACCCTCTAAATGA				
*β-ACT*	MT880275.1	F:GTATGGAATCTTGCGGTATC	76	92.97	0.9996	*y* = 3.5028*x* + 13.025
		R:AGGTCCTTACGGATGTCA				
*RPL32*	MT883789.1	F:CCATCAATCGGATCGCTAT	178	95.74	0.9958	*y* = 3.4283*x* + 15.441
		R:ATTGTGGACCAGGACCTT				
*RPL40*	MT883790.1	F:CAAGCGAAACTGGCGTAA	96	91.43	0.9989	*y* = 3.5461*x* + 14.565
		R:TTTGAACCGTAACCGATG				
*EF1-α*	MT883788.1	F:GCTGATTGTGGGAGTGAA	144	90.37	0.9985	*y* = 3.5767*x* + 13.088
		R:CCAGAAATGGGTACGAAA				
*α-TUB*	MT880274.1	F:GACTCCTTCAACACCTTCTT	98	90.04	0.9981	*y* = 3.5863*x* + 13.786
		R:CGGACCTCATCAACTACAG				
*β-TUB*	MT883787.1	F:CTCAACATCCAGAACAAGAAC	168	88.65	0.9971	*y* = −3.6278*x* + 13.901
		R:GGTGAACTGCTCCGAGAT				
*18S*	MT889641.1	F:TGTCTGCCTTATCAACTTTC	113	89.32	0.9999	*y* = −3.6076*x* + 3.4139
		R:GATGTGGTAGCCGTTTCTCA				
*28S*	MT889642.1	F:CCCGACGCAAGTCAACG	96	85.02	0.9999	*y* = −3.7423*x* + 10.850
		R:GCACAGTCCGAGACAGCAC				
*AK*	MT880273.1	F:CGATGACCACTTCCTGTT	153	86.28	0.9970	*y* = 3.7014*x* + 14.800
		R:ATGGAGATAAGACGGAGATG				
*CSP1*	KT357395.1	F:TCTGCTGGTGCCCTATAT	183	104.16	0.9940	*y* = −3.2262*x* + 21.469
		R:TTCGTGGTTGATGAGATGG				
*SOD*	MT883791.1	F:ATTGAGGCGGATGTTGCT	151	85.41	0.9991	*y* = −3.7295*x* + 17.319
		R:TCTTGCTGAGGTTGTGGC				

Using the obtained sequences of these selected candidate reference genes, gene-specific primers used in the qRT-PCR experiments were designed with Primer Premier 5 software (Premier Biosoft, www.premierbiosoft.com). The primer design parameters were as follows: amplicon size, 76–178 bp; melting temperature, 52°C–62°C; primer length, 17–21 bp; and GC content, 40%–60% (Table 1). All the primers used in this study were synthesized by Sangon Biotechnology Co., Ltd. (Shanghai, China).

### Collection of Insect Samples Under Different Biotic or Abiotic Conditions

All the insect samples collected included three biological replicates. After collection, the samples were flash-frozen in liquid nitrogen and then stored at −80°C for later total RNA extractions. Except for the temperature treatment, the growth environments of all other treatments were the same as in the section “Insects.” All the insects subjected to the treatments were given an adequate food supply except for starvation treatment.

#### Biotic Conditions

##### Developmental Stages

Samples of *A. dissimilis* at the egg stage (400 eggs), larval stage, including first- (100 individuals), second- (50 individuals), third- (20 individuals), fourth- (10 individuals), and fifth- (10 individuals) instar larvae, pupal stage (10 male and 10 female pupae, first day after pupation), and adult stage (10 male and 10 female moths, first day after emergence) were collected.

##### Tissues

The larval tissues, including the head, salivary gland, midgut, Malpighian tubule, fat body, hemolymph, and epidermis, were dissected from fourth-instar larvae of *A. dissimilis* under a dissecting microscope using a dissection needle and tweezer. Adult tissues, such as the antenna, head, thorax, abdomen, leg, and wing, were dissected from 10 males and 10 females (second day after emergence). After dissection, each tissue was washed three times with PBS solution (140 mM NaCl, 2.70 mM KCl, 10 mM Na_2_HPO_4_ and 1.80 mM KH_2_PO_4_, and pH 7.40), and then, pooled in the 1.5-ml RNase-free centrifuge tubes. For each sample, tissues of 20 individuals were dissected and collected.

#### Abiotic Conditions

##### Diets

Fourth-instar larvae of *A. dissimilis* were fed with Chinese cabbage leaf, maize seedlings, wheat seedlings, or artificial diet for 2 days, and then 10 whole individuals per treatment were collected.

##### Insecticide-Induced Stress

Two insecticides, chlorantraniliprole and lambda-cyhalothrin, that are widely used in the control of *A. dissimilis*, were chosen for this experiment. First, the LC_50_ values of the two insecticides to the fourth-instar larvae were measured using the artificial feed mixture method. Each insecticide was diluted with acetone to obtain different concentrations, and then, 10 μl of the diluent in various concentrations was dropped evenly on a piece of artificial diet (0.80 cm × 0.80 cm × 0.50 cm). After the solvent evaporated, the fourth-instar larvae that had been starved for 6 h were individually placed in a clean glass tube and fed the insecticide-treated artificial diet, with acetone as the control. Each treatment was repeated three times, with 24 larvae per replicate. After 48 h, the larval mortality rates of the different treatments were recorded to calculate the LC_50_ (Supplementary Table S2). Subsequently, another batch of fourth-instar larvae that had been starved for 6 h were treated with the LC_50_ value of each insecticide, and after 6, 12, 24, and 48 h, the surviving insects were collected, and each insect sample included 10 individuals.

##### Temperature

Fourth-instar larvae were placed in climatic chambers maintained at 4°C, 27°C, and 40°C. After 2, 6, and 12 h, 10 whole individuals per temperature treatment were collected.

##### Starvation

Fourth-instar larvae were collected after being starved for 12 h and 24 h. In total, 10 whole individuals were included per sample.

### Total RNA Extraction, cDNA Synthesis, and qRT-PCR

The total RNA extraction and cDNA synthesis of each sample were performed as described in the section “Candidate Reference Gene Clones and qRT-PCR Primer Design.” qRT-PCR was performed using an ABI Q3 Real-time PCR System (Applied Biosystems, United States) with SYBR Green SuperReal PreMix Plus RT-PCR Kit (Tiangen Biotech, China) in a final volume of 20 μl, which contained 1 μl of cDNA, 0.6 μl of each primer (10 μM/L) as listed in Table 1, 10.0 μl of 2× SuperReal PreMix Plus, 0.4 μl of 50× ROX Reference Dye, and 7.4 μl of RNase-free ddH_2_O. The qRT-PCR conditions were as follows: 95°C for 15 min, followed by 40 cycles of 95°C for 10 s and 60°C for 32 s. The specificity levels of the primers used here were confirmed by melting curve analyses and 1.5% agarose gel electrophoresis. The melting curves were generated by measuring fluorescence through the dissociation temperature of the PCR product using a temperature transition rate of 0.1°C/s for all the reactions. For each gene, three biological samples were performed, with each sample measured in triplicate. To obtain the amplification efficiency (E), where *E* = [10^(1/−slope)^ − 1] × 100%, and the correlation coefficient of each primer pair, 10-fold dilution series of cDNAs (1:1, 1:10, 1:100, 1:1,000, and 1:10,000) were used as templates to construct a standard curve (Pfaffl, 2001; Pfaffl et al., 2004).

### Gene Expression Stability Analysis

The expression stabilities of the 10 selected candidate reference genes were evaluated using the comparative Δ cycle threshold (Ct) method and three commonly used software programs (geNorm version 3.5, NormFinder version 0.953, and BestKeeper version 1) in all the insect samples under different biotic and abiotic conditions (Vandesompele et al., 2002; Andersen et al., 2004; Pfaffl et al., 2004; Silver et al., 2006; Xie et al., 2012). Finally, a comprehensive tool, RefFinder,^1^ was used to rank the stability order of the selected reference genes (Xie et al., 2012). The detailed calculation method of each statistical algorithm was the same as described previously (Vandesompele et al., 2002; Andersen et al., 2004; Pfaffl et al., 2004; Silver et al., 2006; Xie et al., 2012).

### Validation of the Selected Reference Gene

To validate the stability of the selected reference genes, the mRNA expression levels of a gene encoding a binding chemosensory protein (*CSP1*, GenBank accession no. KT357395.1) and the antioxidant enzyme gene encoding superoxide dismutase (*SOD*, GenBank accession no. MT883791.1) were examined in this study. The *CSP1* transcript levels were assessed at different developmental stages and in different larval tissues (head, fat body, and midgut) of *A. dissimilis* with gene-specific primers listed in Table 1. For the different developmental stages, the expression profiles of *CSP1* were estimated using *EF1-α*, *RPL40*, *18S* (the three most stable reference genes), and *GAPDH* (the least stable reference gene) that were recommend by RefFinder as the reference genes. For the different larval tissues, the expression profiles of *CSP1* were estimated using *RPL40*, *α-TUB*, *RPL32* (the three most stable reference genes), and *β-ACT* (the least stable reference gene) as the reference genes. The *SOD* expression levels were determined in fourth-instar larvae of *A. dissimilis* exposed independently to two insecticides and four different diets using specific primers listed in Table 1. For the insecticide treatments, the expression levels of *SOD* were evaluated using *EF1-α*, *β-ACT*, *GAPDH* (the three most stable reference genes), and *RPL32* (the least stable reference gene) as the reference genes. In contrast, the expression profiles of *SOD* in *A. dissimilis* feeding on different diets were evaluated using *RPL40*, *18S*, *RPL32* (the three most stable reference genes), and *GAPDH* (the least stable reference gene) as the reference genes. The qRT-PCR was performed as described in the section “Total RNA Extraction, cDNA Synthesis, and qRT-PCR,” and the relative expression levels of the two target genes were calculated using the 2^−ΔΔCt^ method (Livak and Schmittgen, 2000). The statistical differences in the target gene expression levels among different treatments were analyzed by a one-way ANOVA with Tukey’s HSD multiple comparisons.

## Results

### Validation of Primer Specificity and qRT-PCR Amplification Efficiency

To validate the primer specificity of the 10 candidate reference genes and two target genes in the qRT-PCR experiment, 1.5% agarose gel electrophoresis and melting curve analyses were performed. The agarose gel electrophoresis of all the PCR products showed single bands of the expected sizes, and no nonspecific amplicons or primer dimmers were observed (Supplementary Figure S2). Additionally, all the melting curves showed single dissociation peaks (Supplementary Figure S3), which confirmed that the primers designed for the selected reference genes were highly specific and could be used for further qRT-PCR analyses. The amplification efficiency levels of all the primer pairs ranged from 85.02% (*28S*) to 104.16% (*CSP1*), and the correlation coefficients were all greater than 0.990 (*p* < 0.01), ranging from 0.994 (*CSP1*) to 0.9999 (*18S* and *28S*; Table 1), which reflected their stability and specificity.

### Transcriptional Profiles of the Candidate Reference Genes

**Figure 1:**
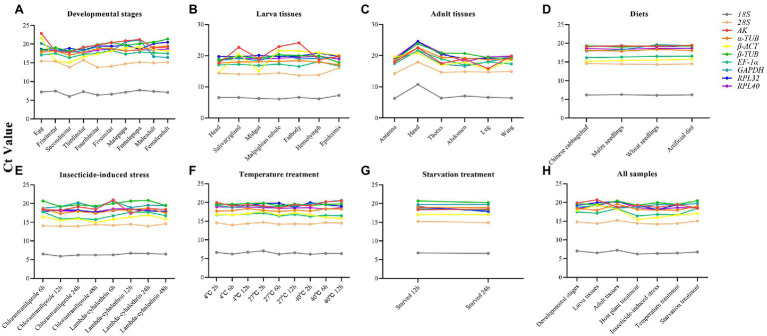
Variation in the reference genes expression using the distribution of Ct values in line charts. **(A)** Developmental stages; **(B)** Larvae tissues; **(C)** Adult tissues; **(D)** Diets; **(E)** Insecticides; **(F)** Temperature; **(G)** Starvation; **(H)** All samples.

The expression profiles of the candidate reference genes in different insect samples were evaluated by comparing the Ct values. The Ct values of these reference genes varied widely, ranging from 6.03 (*18S*) to 24.57 (*RPL40*; Supplementary Table S3), for all the experimental conditions (Figure 1). The *18S* gene showed the greatest expression levels, with the lowest Ct values, whereas the *AK* gene showed the lowest levels. The expression levels of each gene under different conditions revealed that most of these genes remained stable under different conditions, but some genes varied widely, such as *AK* in larval tissues (Figure 1).

### Expression Stability of Candidate Reference Genes Under Biotic Conditions

#### Developmental Stages

**Figure 2:**
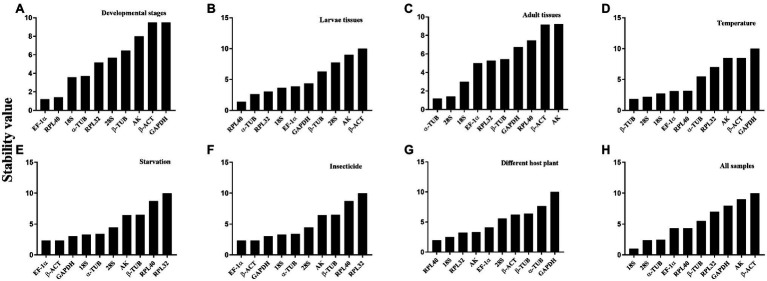
Expression stability ranking order of the candidate reference genes for *A. dissimilis* at different conditions according to ReFinder. A lower-ranking value of the gene denotes it has more stable expression stability. **(A)** Developmental stages; **(B)** Larvae tissues; **(C)** Adult tissues; **(D)** Temperature; **(E)** Starvation; **(F)** Insecticides; **(G)** Diets; **(H)** All samples.

**Figure 3:**
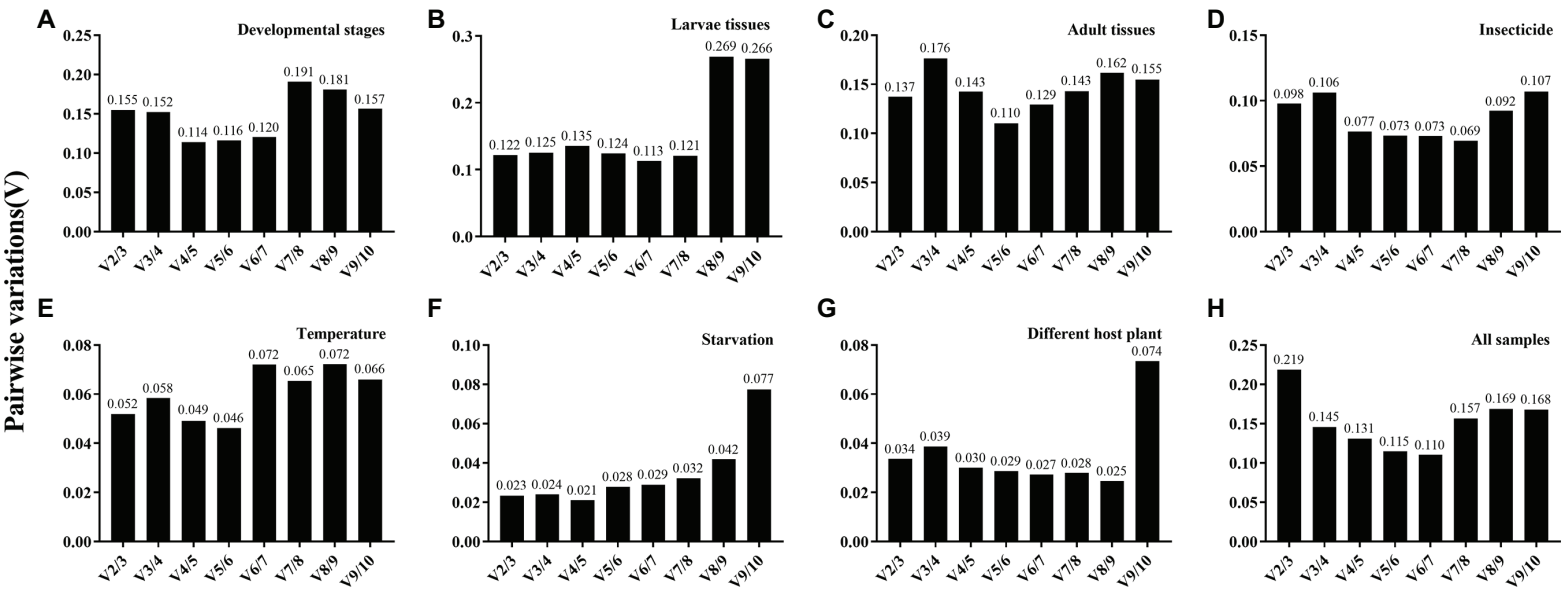
Pairwise variation (V) analysis of the candidate reference genes in *A. dissimilis* under different conditions. Pairwise variation analysis is performed by geNorm to determine the optimal number of reference genes for normalization in different conditions. Each pairwise variation value is compared with 0.15, and when V*_n_*/V_*n*+1_ below 0.15, the optimal number of reference genes is *n*. **(A)** Developmental stages; **(B)** Larvae tissues; **(C)** Adult tissues; **(D)** Insecticides; **(E)** Temperature; **(F)** Starvation; **(G)** Diets; **(H)** All samples.

The overall stability ranking orders recommended by geNorm, NormFinder, and ΔCt methods were similar, showing *EF1-α* and *RPL40* as the two most stable genes, and *GAPDH* and *β-ACT* as the least stable genes. The ranking order determined by BestKeeper showed *GAPDH* and *RPL32* being the most stable genes and *AK* and *β-ACT* being the least stable genes (Table 2). The stability analysis of RefFinder ranked the genes from most stable to least stable as follows: *EF1-α* > *RPL40* > *18S* > *α-TUB* > *RPL32* > *28S* > *β-TUB* > *AK* > *β-ACT* > *GAPDH* (Figure 2A). The geNorm analysis showed that the pairwise variation values of V_4/5_, V_5/6_, and V_6/7_ were all less than 0.15 (Figure 3A). On the basis of cost and convenience, according to the geNorm manual, we selected three as the optimal number of reference genes. Consequently, the combination of *EF1-α*, *RPL40*, and *18S*, the three most stable genes, was recommended as being suitable for normalizing qRT-PCR data at different developmental stages of *A. dissimilis* (Table 3).

**Table 2:** Expression stability of the candidate reference genes in *A. dissimilis* under different biotic conditions.

Biotic condition	Reference gene	geNorm		Normfinder		Bestkeeper		ΔCt	
		Stability	Rank	Stability	Rank	Stability	Rank	Stability	Rank
Developmental stages	*18S*	0.66	5	0.32	3	0.74	8	0.52	3
	*28S*	0.73	6	0.53	5	0.50	3	0.71	5
	*AK*	0.95	7	0.81	8	0.79	9	1.54	8
	*EF1-α*	0.44	1	0.31	1	0.64	7	0.51	2
	*GAPDH*	1.25	9	1.07	9	0.48	2	1.58	9
	*RPL32*	0.58	3	0.59	6	0.33	1	0.76	6
	*RPL40*	0.44	1	0.32	2	0.56	5	0.29	1
	*α-TUB*	0.49	2	0.42	4	0.62	6	0.71	4
	*β-ACT*	1.12	8	1.08	10	0.84	10	1.93	10
	*β-TUB*	0.61	4	0.64	7	0.54	4	1.00	7
Larval tissues	*18S*	0.58	4	0.54	6	0.07	2	0.39	1
	*28S*	0.80	7	0.77	8	0.05	1	0.78	7
	*AK*	1.17	8	1.52	9	0.79	9	2.51	9
	*EF1-α*	0.40	2	0.51	5	0.16	4	0.52	5
	*GAPDH*	0.66	5	0.24	2	0.75	8	0.72	6
	*RPL32*	0.37	1	0.54	7	0.16	3	0.43	4
	*RPL40*	0.37	1	0.21	1	0.59	7	0.40	2
	*α-TUB*	0.47	3	0.38	3	0.29	5	0.42	3
	*β-ACT*	1.50	9	1.83	10	0.97	10	3.02	10
	*β-TUB*	0.72	6	0.44	4	0.55	6	0.92	8
Adult tissues	*18S*	0.41	2	0.30	3	0.97	7	1.74	3
	*28S*	0.35	1	0.29	2	0.98	10	1.34	1
	*AK*	1.05	8	0.90	9	0.86	3	2.31	10
	*EF1-α*	0.66	4	0.49	5	0.91	5	1.75	5
	*GAPDH*	0.90	7	0.67	8	0.85	2	1.74	4
	*RPL32*	0.70	5	0.46	4	0.97	8	2.19	8
	*RPL40*	0.78	6	0.66	7	0.93	6	2.26	9
	*α-TUB*	0.35	1	0.19	1	0.98	9	1.52	2
	*β-ACT*	1.18	9	1.06	10	0.70	1	1.92	7
	*β-TUB*	0.58	3	0.61	6	0.90	4	1.90	6

**Table 3:** Suitable reference genes recommended for *A. dissimilis* at different experimental conditions.

Factors	Experimental conditions	Recommended reference genes		
Biotic factors	Developmental stages	*EF1-α*	*RPL40*	*18S*
	Larval tissues	*RPL40*	*α-TUB*	
	Adult tissues	*α-TUB*	*28S*	
Abiotic factors	Diets	*RPL40*	*18S*	
	Insecticide treatment	*EF1-α*	*β-ACT*	
	Temperature treatment	*β-TUB*	*28S*	
	Starvation treatment	*EF1-α*	*β-ACT*	
All samples		*18S*	*28S*	*α-TUB*

#### Larval Tissues

For different larval tissues, all four programs identified *β*-*ACT* and *AK* as the least stable genes. *RPL32* and *RPL40* were identified as the most stable genes by geNorm. NormFinder, BestKeeper, and the ΔCt method identified *RPL40*, *28S*, and *18S* as the most stable gene, respectively (Table 2). RefFinder ranked the candidate reference genes from high to low stability as follows: *RPL40* > *α-TUB* > *RPL32* > *18S* > *EF1-α* > *GAPDH* > *β-TUB* > *28S* > *AK* > *β-ACT* (Figure 2B). The pairwise variation showed that two reference genes were suitable for normalizing gene expression in different larval tissues of *A. dissimilis* because the value of V_2/3_ was less than 0.15 (Figure 3B; Table 3).

#### Adult Tissues

In the adult tissue assessment, *28S*, *α-TUB*, and *18S* were identified as the top three stable genes by all the programs, except for BestKeeper, which identified the three best-suited genes as *β-ACT*, *GAPDH*, and *AK*. The least stable genes were identified by NormFinder and geNorm as *AK* and *β-ACT*, by BestKeeper as *28S* and *α-TUB*, and by the ΔCt method as *AK* and *RPL40* (Table 2). RefFinder ranked the stability order as follows: *α-TUB* > *28S* > *18S* > *EF1-α* > *RPL32* > *β-TUB* > *GAPDH* > *RPL40* > *β-ACT* > *AK* (Figure 2C). The pairwise variation value of V_2/3_ in the geNorm analysis was less than 0.15 (Figure 3C). Thus, the combination of *α-TUB* and *28S* was suitable for normalizing qRT-PCR data in adult tissue samples of *A. dissimilis* (Table 3).

#### Dietary Treatments

Both geNorm and the ΔCt method ranked *18S*, *RPL32*, and *28S* as the top three reference genes for *A. dissimili* receiving different dietary treatments. NormFinder placed *RPL40* as the most stable gene, whereas BestKeeper identified *28S* as the most stable genes. In addition, *GAPDH* was identified as the least stable gene by all the methods, except BestKeeper, which identified *EF1-α* as the least stable gene (Table 2). The ranking of the most stable genes by the RefFinder analysis was as follows: *RPL40* > *18S* > *RPL32* > *AK* > *EF1-α* > *28S* > *β-ACT* > *β-TUB* > *α-TUB* > *GAPDH* (Figure 2G), and the pairwise variation analysis showed that the value of V_2/3_ was less than 0.15 (Figure 3G). Thus, *RPL40* and *18S* are appropriate to normalize the gene expression profiles in *A. dissimili* larvae fed on different diets (Table 3).

### Expression Stability of Candidate Reference Genes Under Abiotic Conditions

#### Insecticide Treatments

The stability analyses performed by geNorm, NormFinder, and ΔCt algorithms identified *AK*, *GAPDH*, and *EF1-α* as the least stable genes, although their rank orders were different. The geNorm analysis inferred that *α-TUB* and *β-TUB* were the most stable genes, whereas NormFinder, BestKeeper, and the ΔCt method identified *RPL40*, *28S*, and *RPL32* as the most stable gene, respectively (Table 4). The RefFinder software ranked the expression stability of the reference genes as follows: *EF1-α* > *β-ACT* > *GAPDH* > *18S* > *α-TUB* > *28S* > *AK* > *β-TUB* > *RPL40* > *RPL32* (Figure 2D). The pairwise variation analysis showed that the value of V_2/3_ was less than 0.15 (Figure 3D). Thus, the two reference genes *EF1-α* and *β-ACT* are sufficient to normalize gene expression under these experimental conditions (Table 3).

**Table 4:** Expression stability of the candidate reference genes in *A. dissimilis* under different abiotic conditions.

Abiotic condition	Reference gene	geNorm		Normfinder		Bestkeeper		ΔCt	
		Stability	Rank	Stability	Rank	Stability	Rank	Stability	Rank
Diets	*18S*	0.07	1	0.13	8	0.77	3	0.07	1
	*28S*	0.09	2	0.16	9	0.66	1	0.12	3
	*AK*	0.13	3	0.06	3	0.92	9	0.16	5
	*EF1-α*	0.16	5	0.03	2	0.99	10	0.19	7
	*GAPDH*	0.31	9	0.51	10	0.76	2	0.80	10
	*RPL32*	0.07	1	0.13	7	0.80	5	0.12	2
	*RPL40*	0.14	4	0.01	1	0.88	8	0.12	4
	*α-TUB*	0.20	8	0.13	6	0.87	7	0.21	8
	*β-ACT*	0.17	6	0.09	4	0.79	4	0.26	9
	*β-TUB*	0.19	7	0.12	5	0.85	6	0.18	6
Insecticide	*18S*	0.37	3	0.14	2	0.82	7	0.26	2
	*28S*	0.46	6	0.34	7	0.15	1	0.27	3
	*AK*	0.69	9	0.74	10	0.32	2	1.04	10
	*EF1-α*	0.50	7	0.47	8	0.84	8	0.86	9
	*GAPDH*	0.59	8	0.48	9	0.38	3	0.68	8
	*RPL32*	0.43	5	0.24	4	0.46	4	0.25	1
	*RPL40*	0.39	4	0.11	1	0.72	5	0.31	4
	*α-TUB*	0.21	1	0.20	3	0.91	10	0.57	5
	*β-ACT*	0.28	2	0.28	5	0.82	6	0.62	6
	*β-TUB*	0.21	1	0.31	6	0.88	9	0.67	7
Temperature	*18S*	0.13	1	0.12	3	0.89	10	0.30	6
	*28S*	0.20	3	0.03	1	0.79	7	0.24	3
	*AK*	0.45	8	0.40	8	0.46	4	0.60	9
	*EF1-α*	0.13	1	0.15	6	0.83	8	0.28	5
	*GAPDH*	0.51	9	0.45	10	0.28	2	0.63	10
	*RPL32*	0.33	6	0.35	7	−0.05	1	0.41	7
	*RPL40*	0.23	4	0.13	4	0.42	3	0.18	1
	*α-TUB*	0.25	5	0.14	5	0.68	6	0.28	4
	*β-ACT*	0.39	7	0.44	9	0.46	5	0.58	8
	*β-TUB*	0.16	2	0.04	2	0.89	9	0.22	2
Starvation	*18S*	0.08	3	0.03	3	0.84	5	0.11	5
	*28S*	0.14	6	0.03	2	0.91	7	0.23	7
	*AK*	0.11	5	0.24	8	−0.16	1	0.11	4
	*EF1-α*	0.09	4	0.01	1	0.94	9	0.14	6
	*GAPDH*	0.03	1	0.16	7	0.35	2	0.03	2
	*RPL32*	0.33	9	0.54	10	0.92	8	0.91	10
	*RPL40*	0.22	8	0.19	8	0.86	6	0.47	9
	*α-TUB*	0.06	2	0.09	5	0.83	4	0.06	3
	*β-ACT*	0.03	1	0.14	6	0.38	3	0.01	1
	*β-TUB*	0.17	7	0.04	4	0.97	10	0.32	8

#### Temperature Treatment

The top three least stable genes determined by geNorm, NormFinder, and the ΔCt method were *GAPDH*, *AK*, and *β-ACT*, whereas the determination by BestKeeper was *18S*, *β-TUB*, and *EF1-α*. GeNorm identified *18S* and *EF1-α* as the most stable genes. NormFinder, BestKeeper, and the ΔCt method identified *28S*, *RPL32*, and *RPL40* as the most stable gene, respectively (Table 4). The stability ranking of the reference genes from the most stable to least stable gene by the RefFinder analysis was as follows: *β-TUB* > *28S* > *18S* > *EF1-α* > *RPL40* > *α-TUB* > *RPL32* > *AK* > *β-ACT* > *GAPDH* (Figure 2E). The pairwise variation values were all less than 0.15 (Figure 3E). Thus, *β-TUB* and *28S* are sufficient to normalize qRT-PCR data from the temperature-treated samples (Table 3).

#### Starvation Treatment

The top three ranked reference genes as determined by geNorm and the ΔCt method for insect samples after starvation were *β-ACT*, *GAPDH*, and *α-TUB*. NormFinder identified *EF1-α*, *28S*, and *18S* as the top three suitable reference genes, whereas BestKeeper identified *AK*, *GAPDH*, and *β-ACT*. The least stable gene identified by geNorm, NormFinder, and the ΔCT method was *RPL32*, whereas *β-TUB* was identified by BestKeeper (Table 4). The reference gene stability ranking, from most to least stable, as determined by the RefFinder analysis was as follows: *EF1-α* > *β-ACT* > *GAPDH* > *18S* > *α-TUB* > *28S* > *AK* > *β-TUB* > *RPL40* > *RPL32* (Figure 2F). The pairwise variation analysis showed that all the values were less than 0.15 (Figure 3F). Therefore, *EF1-α* and *β-ACT* are appropriate for normalizing gene expression data collected under starved conditions (Table 3).

### Ranking of *Athetis dissimilis* Candidate Reference Genes Across All the Samples

For all the samples, the stability ranking results determined by geNorm were similar to those obtained by the ΔCt method, which identified *28S*, *18S*, and *α-TUB* as the three most stable genes. NormFinder identified *18S*, *α-TUB*, and *RPL40* as the three most stable genes, whereas BestKeeper selected *GAPDH*, *AK*, and *β-ACT* as the most appropriate candidate genes (Table 5). The RefFinder analysis ranked the candidate reference genes from the most stable to the least stable as follows: *18S* > *28S* > *α-TUB* > *EF1-α* > *RPL40* > *β-TUB* > *RPL32* > *GAPDH* > *AK* > *β-ACT* (Figure 2H). The geNorm analysis determined that the pairwise variation values of V_3/4_ were less than 0.15 (Figure 3H). Thus, *18S*, *28S*, and *α-TUB* are recommended as the most stable reference genes for normalizing qRT-PCR data from all the samples (Table 3).

**Table 5:** Expression stability of the candidate reference genes in *A. dissimilis* under all samples.

Reference gene	geNorm		Normfinder		Bestkeeper		ΔCt	
	Stability	Rank	Stability	Rank	Stability	Rank	Stability	Rank
*18S*	0.37	1	0.30	1	0.85	10	0.75	2
*28S*	0.37	1	0.43	4	0.71	4	0.70	1
*AK*	1.08	8	0.96	9	0.61	2	1.62	9
*EF1-α*	0.62	3	0.44	5	0.78	8	0.98	4
*GAPDH*	0.91	7	0.79	8	0.57	1	1.11	8
*RPL32*	0.77	6	0.52	7	0.75	5	1.09	7
*RPL40*	0.72	5	0.42	3	0.79	9	1.05	6
*α-TUB*	0.58	2	0.38	2	0.77	7	0.86	3
*β-ACT*	1.23	9	1.15	10	0.66	3	2.00	10
*β-TUB*	0.67	4	0.51	6	0.76	6	0.97	5

### Target Gene Validation Using the Selected Reference Genes

**Figure 4:**
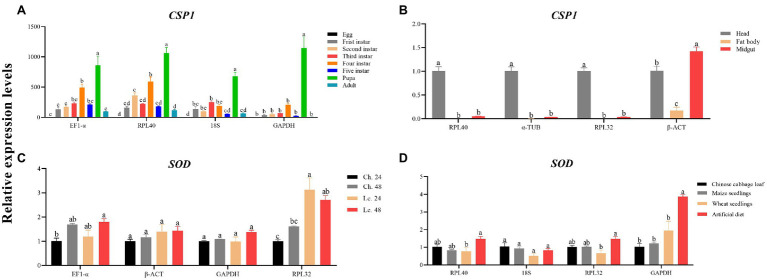
Validation of the candidate reference genes suitable for *A. dissimilis*. **(A)** *CSP1* relative expression levels of *A. dissimilis* at different developmental stages. **(B)** *CSP1* relative expression levels of *A. dissimilis* in three different larval tissues. **(C)** *SOD* relative expression levels of *A. dissimilis* treated with different insecticides (Ch.: chlorantraniliprole, Lc.: lambda-cyhalothrin) for various times. **(D)** *SOD* relative expression levels of *A. dissimilis* feeding on different diets. Values are means ± SEM. Different letters indicate significant differences (*p* < 0.05, one-way ANOVA followed by Tukey’s HSD multiple comparison).

The transcript levels of *CSP1* and *SOD* were assessed under various experimental conditions to verify the performance levels of the selected reference genes. For developmental stages, the qRT-PCR results using either *EF1-α* and *RPL40* or *18S* as normalizers were more consistent than those using *GAPHD* as the normalizer (Figure 4A). The same results were achieved using larval tissues (Figure 4B). Furthermore, for the insecticide treatments, the expression profile of *SOD* clearly exhibited differences when *RPL32* was used in the normalization (Figure 4C). Moreover, for different dietary treatments, the results were much different using *RPL40* and *18S* or *RPL32* as reference gene(s) compared with using *GAPDH* (Figure 4D).

## Discussion

Quantitative real-time PCR is an important method in gene expression research that uses reference genes as standards to calibrate the expression levels of target genes. Therefore, it is essential to select the appropriate reference genes. At present, reference genes have been screened in a variety of insects (Zhang et al., 2015; Yin et al., 2020; Xie et al., 2021), but stable reference genes for *A. dissimilis* under various conditions remain undetermined. Recently, the transcriptomes of different tissues of *A. dissimilis* were sequenced (Dong et al., 2016; Sun et al., 2016; Liu et al., 2019), indicating that more genes can be studied in depth. Therefore, it is urgent to identify reference genes that are stably expressed under different experimental conditions.

In this study, four commonly used tools, geNorm, NormFinder, BestKeeper, and ΔCt, were used to analyze the expression stability levels of 10 candidate reference genes (*18S*, *28S*, *AK*, *EF1-α*, *GAPDH*, *RPL32*, *RPL40*, *α-TUB*, *β-ACT*, and *β-TUB*) under different biotic and abiotic conditions. Because the four tools may determine different gene stability levels owing to the use of different algorithms, the web-based analysis tool RefFinder, which integrates all four major calculation programs, was used to evaluate and screen the optimal reference genes.

The optimal number of reference genes can be determined through the geNorm analysis by using the paired mutation value (V_*n*/*n + 1*_; Vandesompele et al., 2002). The results showed that *EF1-α*, *RPL40*, and *18S* are suitable for the normalization of data from different developmental stages, *RPL40* and *α-TUB* for larval tissues, *α-TUB* and *28S* for adult tissues, *RPL40* and *18S* for dietary treatments, *EF1-α* and *β-ACT* for insecticide treatments, *β-TUB* and *28S* for temperature treatments, *EF1-α* and *β-ACT* for starvation treatments, and *18S*, *28S*, and *α-TUB* for all the samples.

Elongation factor 1 alpha was identified as the most stable reference gene of *A. dissimilis* in different developmental stages and insecticide treatments. In translation, *EF1-α* encodes a protein that catalyzes the GTP-dependent binding of aminoacyl tRNA to the acceptor site of the ribosome (Ponton et al., 2011). Consequently, it is one of the most abundant proteins in cells and is highly conserved among species (Xie et al., 2021). *EF1-α* is regarded as a suitable reference gene in other organisms under different conditions, such as *Spodoptera litura* at different temperatures (Lepidoptera: Noctuidae; Lu et al., 2013), *Locusta migratoria* at different developmental stages (Orthoptera: Acrididae; Yang et al., 2014), *Frankliniella occidentalis* (Thysanoptera: Thripidae; Zheng et al., 2014), and *Hippodamia convergens* (Coleoptera: Coccinellidae; Pan et al., 2015) under different developmental stages and temperature treatments, and *Sesamia inferens* in different tissues and developmental stages (Lepidoptera: Noctuidae; Sun et al., 2015). Additionally, *EF1-α* is the first in the reference ranking provided by the ICG website (Sang et al., 2018).

Here, *RPL40* was considered as the most stable reference gene for dietary treatments and larval tissues. The protein encoded by *RPL40* forms the structure of the ribosome and plays very important roles in cell life activities. Many genes in the RPL family are used as reference genes for stable expression in insects, such as *Solenopsis invicta* (Hymenoptera, Formicidae; Cheng et al., 2013), *Anastrepha obliqua* (Diptera, Tephritidae; Nakamura et al., 2016), and *Cimex lectularius* (Hemiptera, Cimicidae; Mamidala et al., 2011). On the ICG website, RPL was ranked as the fifth most stable reference gene.

Alpha-tubulin and *β-TUB* are the most stable reference genes expressed in the adult tissues and temperature treatments of *A. dissimilis*. *TUB* belongs to the structural gene family of eukaryotes, helping to form the basic components of microtubules and skeletons, and it regulates cell division, shape, movement, and intracellular activities (Nielsen et al., 2010). It has also been used as reference gene in many organisms (Huis et al., 2010; Kozera and Rapacz, 2013). In the reference ranking provided by the ICG website, the *TUB* family ranked sixth. *TUB* is also used as the best reference gene in insects under many conditions, such as the geographic populations of *Nilaparvata lugens* (Hemiptera: Delphacidae; Miao et al., 2014), developmental stages and temperature treatments of *Sogatella furcifera* (Hemiptera: Delphacidae; An et al., 2016), and temperature treatments of *Bemisia tabaci* (Dai et al., 2017).

Before this study, *GAPDH* was used as the reference gene when studying the gene expression of adult *A. dissimilis* (Sun et al., 2016; Liu et al., 2019). However, it is not an appropriate choice, and this study provides a basis for selecting appropriate reference genes.

RefFinder recommended *18S*, *28S*, and *α-TUB* as the most stable reference genes for *A. dissimilis* for all the tested samples. Ribosomal RNAs (rRNAs), including 18S rRNA and 28S rRNA, mainly participate in protein synthesis and are highly expressed in all biological cells. Because the RNA polymerase that synthesizes rRNA is different from the RNA polymerase that synthesizes mRNA, and the regulation of rRNA synthesis is not related to the mRNA level, rRNA has been regarded as an ideal reference gene (Bustin, 2000), such as in different tissues of *Rhodnius prolixus* (Hemiptera, Reduviidae; Paim et al., 2012) and in different body parts of *N. lugens* (Hemiptera: Delphacidae; Miao et al., 2014). *18S* ranked eighth in the reference ranking provided by the ICG website. Although *18S* is identified as the most stable reference gene under all simples in *A. dissimilis*, the expression levels of *18S* were much higher than the target genes *SOD* and *CSP1* used for verification, the high expression may mask our correct understanding of the actual expression of target genes, and the same goes for the *28S*. Therefore, in future research, we should consider this issue and further consider the selection of reference genes according to the expression of our target genes.

An unstable reference gene is insufficient to normalize the gene expression data or may generate the wrong interpretation. According to the results of the present research, the three most stable genes and one least stable gene were used for the normalization of the expression levels of *CSP1* and *SOD* in developmental stages, larval tissues, insecticide treatments, and dietary treatments of *A. dissimilis* to validate their stability. The result shows that unstable reference gene is insufficient to normalize the gene expression data or may generate the wrong interpretation Therefore, the selection and validation of the best reference genes are crucial to determine the accuracy of the expression patterns of different genes in *A. dissimilis*. This will benefit to the future studies on gene functions in *A. dissimilis* and other insects and will facilitate the generation of more reliable and accurate data on gene expression in *A. dissimilis*.

## Data Availability Statement

The original contributions presented in the study are included in the article/**Supplementary Material**, further inquiries can be directed to the corresponding authors.

## Author Contributions

JT, XG, and MZ conceived and designed the research. JT and SD conducted the experiments. JT and SS analyzed the data. JT, GL, and MZ wrote the article. All authors have read and agreed to the published version of the article.

## Funding

This research was supported by the National Key Research and Development Program of China (Nos. 2018YFD0200600 and 2017YFD0201700) and the National Natural Science Foundation of China (No. 31801735).

## Conflict of Interest

The authors declare that the research was conducted in the absence of any commercial or financial relationships that could be construed as a potential conflict of interest.

## Publisher’s Note

All claims expressed in this article are solely those of the authors and do not necessarily represent those of their affiliated organizations, or those of the publisher, the editors and the reviewers. Any product that may be evaluated in this article, or claim that may be made by its manufacturer, is not guaranteed or endorsed by the publisher.

## Footnotes

^1^ https://www.heartcure.com.au/reffinder/

## References

[ref1] An X. K. Hou M. L. Liu Y. D. (2016). Reference gene selection and evaluation for gene expression studies using qRT-PCR in the white-backed planthopper, *Sogatella furcifera* (Hemiptera: Delphacidae). J. Econ. Entomol. 109, 879–886. doi: 10.1093/jee/tov333, PMID: 26612891

[ref2] Andersen C. L. Jensen J. L. Ørntoft T. F. (2004). Normalization of real-time quantitative reverse transcription-PCR data: a model-based variance estimation approach to identify genes suited for normalization, applied to bladder and colon cancer data sets. Cancer Res. 64, 5245–5250. doi: 10.1158/0008-5472.CAN-04-0496, PMID: 15289330

[ref3] Brym P. Rusc A. Kaminski S. (2013). Evaluation of reference genes for qRT-PCR gene expression studies in whole blood samples from healthy and leukemia-virus infected cattle. Vet. Immunol. Immunopathol. 153, 302–307. doi: 10.1016/j.vetimm.2013.03.004, PMID: 23548864

[ref4] Bustin S. (2000). Absolute quantification of mRNA using real-time reverse transcription polymerase chain reaction assays. J. Mol. Endocrinol. 25, 169–193. doi: 10.1677/jme.0.0250169, PMID: 11013345

[ref6] Bustin S. Benes V. Nolan T. Pfaffl M. W. (2005). Quantitative real-time RT-PCR--a perspective. J. Mol. Endocrinol. 34, 597–601. doi: 10.1677/jme.1.01755, PMID: 15956331

[ref7] Che Z. Tian Y. Yang J. Liu S. Jiang J. Hu M.. (2019). Screening of insecticidal activity of podophyllotoxin analogues against *Athetis dissimilis*. Nat. Prod. Commun. 14, 117–120. doi: 10.1177/1934578X1901400131

[ref8] Cheng D. Zhang Z. He X. Liang G. (2013). Validation of reference genes in *Solenopsis invicta* in different developmental stages, castes and tissues. PLoS One 8:e57718. doi: 10.1371/journal.pone.0057718, PMID: 23469057 PMC3585193

[ref9] Dai T. M. Lu Z. C. Liu W. X. Wan F. H. (2017). Selection and validation of reference genes for qRT-PCR analysis during biological invasions: the thermal adaptability of *Bemisia tabaci* MED. PLoS One 12:e0173821. doi: 10.1371/journal.pone.0173821, PMID: 28323834 PMC5360248

[ref10] Derveaux S. Vandesompele J. Hellemans J. (2010). How to do successful gene expression analysis using real-time PCR. Methods 50, 227–230. doi: 10.1016/j.ymeth.2009.11.001, PMID: 19969088

[ref11] Dong J. Song Y. Li W. Shi J. Wang Z. (2016). Identification of putative chemosensory receptor genes from the *Athetis dissimilis* antennal transcriptome. PLoS One 11:e0147768. doi: 10.1371/journal.pone.0147768, PMID: 26812239 PMC4727905

[ref12] Fleige S. Pfaffl M. W. (2006). RNA integrity and the effect on the real-time qRT-PCR performance. Mol. Asp. Med. 27, 126–139. doi: 10.1016/j.mam.2005.12.003, PMID: 16469371

[ref13] Fu W. Xie W. Zhang Z. Wang S. Wu Q. Liu Y.. (2013). Exploring valid reference genes for quantitative real-time PCR analysis in *Plutella xylostella* (Lepidoptera: Plutellidae). Int. J. Biol. Sci. 9, 792–802. doi: 10.7150/ijbs.5862, PMID: 23983612 PMC3753443

[ref14] Gao X. K. Zhang S. Luo J. Y. Wang C. Y. Lu L. M. Zhang L. J.. (2017). Identification and validation of reference genes for gene expression analysis in *Aphidius gifuensis* (hymenoptera: Aphidiidae). PLoS One 12:e0188477. doi: 10.1371/journal.pone.0188477, PMID: 29190301 PMC5708624

[ref15] Glare E. M. Divjak M. Bailey M. J. Walters E. H. (2002). β-Actin and GAPDH housekeeping gene expression in asthmatic airways is variable and not suitable for normalising mRNA levels. Thorax 57, 765–770. doi: 10.1136/thorax.57.9.765, PMID: 12200519 PMC1746418

[ref16] Guenin S. Mauriat M. Pelloux J. Van Wuytswinkel O. Bellini C. Gutierrez L. (2009). Normalization of qRT-PCR data: the necessity of adopting a systematic, experimental conditions-specific, validation of references. J. Exp. Bot. 60, 487–493. doi: 10.1093/jxb/ern305, PMID: 19264760

[ref17] Guo H. Jiang L. Xia Q. (2016). Selection of reference genes for analysis of stress-responsive genes after challenge with viruses and temperature changes in the silkworm *Bombyx mori*. Mol. Gen. Genomics. 291, 999–1004. doi: 10.1007/s00438-015-1125-4, PMID: 26437927

[ref18] Guo T. T. Li L. L. Men X. Y. Lu Z. B. Chen H. Wang Z. Y.. (2017). Impact of temperature on the growth and development of *Athetis dissimilis* (Lepidoptera: Noctuidae). J. Econ. Entomol. 110, 274–281. doi: 10.1093/jee/tow229, PMID: 28011680

[ref19] Hu Y. Fu H. Qiao H. Sun S. Zhang W. Jin S.. (2018). Validation and evaluation of reference genes for quantitative real-time PCR in *Macrobrachium nipponense*. Int. J. Mol. Sci. 19:2258. doi: 10.3390/ijms19082258, PMID: 30071669 PMC6121487

[ref20] Huggett J. Dheda K. Bustin S. Zumla A. (2005). Real-time RT-PCR normalisation; strategies and considerations. Genes Immun. 6, 279–284. doi: 10.1038/sj.gene.6364190, PMID: 15815687

[ref21] Huis R. Hawkins S. Neutelings G. (2010). Selection of reference genes for quantitative gene expression normalization in flax (*Linum usitatissimum* L.). BMC Plant Biol. 10:71. doi: 10.1186/1471-2229-10-71, PMID: 20403198 PMC3095345

[ref22] Janska A. Hodek J. Svoboda P. Zamecnik J. Prasil I. T. Vlasakova E.. (2013). The choice of reference gene set for assessing gene expression in barley (*Hordeum vulgare* L.) under low temperature and drought stress. Mol. Gen. Genomics. 288, 639–649. doi: 10.1007/s00438-013-0774-4, PMID: 23979536

[ref23] Kozera B. Rapacz M. (2013). Reference genes in real-time PCR. J. Appl. Genet. 54, 391–406. doi: 10.1007/s13353-013-0173-x, PMID: 24078518 PMC3825189

[ref24] Liu X. L. Sun S. J. Khuhro S. A. Elzaki M. E. A. Yan Q. Dong S. L. (2019). Functional characterization of pheromone receptors in the moth *Athetis dissimilis* (Lepidoptera: Noctuidae). Pestic. Biochem. Physiol. 158, 69–76. doi: 10.1016/j.pestbp.2019.04.011, PMID: 31378363

[ref25] Livak K. Schmittgen T. (2000). Analysis of relative gene expression data using real-time quantitative PCR and the 2-△△Ct method. Methods 25, 402–408. doi: 10.1006/meth.2001.1262, PMID: 11846609

[ref26] Lu Y. Yuan M. Gao X. Kang T. Zhan S. Wan H.. (2013). Identification and validation of reference genes for gene expression analysis using quantitative PCR in *Spodoptera litura* (Lepidoptera: Noctuidae). PLoS One 8:e68059. doi: 10.1371/journal.pone.0068059, PMID: 23874494 PMC3706614

[ref27] Ma K. S. Li F. Liang P. Z. Chen X. W. Liu Y. Gao X. W. (2016). Identification and validation of reference genes for the normalization of gene expression data in qRT-PCR analysis in *Aphis gossypii* (Hemiptera: Aphididae). J. Insect Sci. 16:17. doi: 10.1093/jisesa/iew003, PMID: 28076279 PMC5778981

[ref28] Mahanty A. Purohit G. K. Mohanty S. Nayak N. R. Mohanty B. P. (2017). Suitable reference gene for quantitative real-time PCR analysis of gene expression in gonadal tissues of minnow *Puntius sophore* under high-temperature stress. BMC Genomics 18:617. doi: 10.1186/s12864-017-3974-1, PMID: 28810828 PMC5557063

[ref29] Mamidala P. Rajarapu S. P. Jones S. C. Mittapalli O. (2011). Identification and validation of reference genes for quantitative real-time polymerase chain reaction in *Cimex lectularius*. J. Med. Entomol. 48, 947–951. doi: 10.1603/ME10262, PMID: 21845960

[ref30] Miao Y. Lu Y. Zhu X. Wan H. Shakeel M. Zhan S.. (2014). Selection and evaluation of potential reference genes for gene expression analysis in the brown planthopper, *Nilaparvata lugens* (Hemiptera: Delphacidae) using reverse-transcription quantitative PCR. PLoS One 9:e86503. doi: 10.1371/journal.pone.0086503, PMID: 24466124 PMC3900570

[ref31] Nakamura A. M. Chahad-Ehlers S. Lima A. L. Taniguti C. H. Sobrinho I. Jr. Torres F. R.. (2016). Reference genes for accessing differential expression among developmental stages and analysis of differential expression of OBP genes in *Anastrepha obliqua*. Sci. Rep. 6:17480. doi: 10.1038/srep17480, PMID: 26818909 PMC4730201

[ref32] Nelissen K. Smeets K. Mulder M. Hendriks J. J. Ameloot M. (2010). Selection of reference genes for gene expression studies in rat oligodendrocytes using quantitative real time PCR. J. Neurosci. Methods 187, 78–83. doi: 10.1016/j.jneumeth.2009.12.018, PMID: 20036692

[ref33] Nielsen M. G. Gadagkar S. R. Gutzwiller L. (2010). Tubulin evolution in insects: gene duplication and subfunctionalization provide specialized isoforms in a functionally constrained gene family. BMC Evol. Biol. 10:113. doi: 10.1186/1471-2148-10-113, PMID: 20423510 PMC2880298

[ref34] Paim R. M. Pereira M. H. Di Ponzio R. Rodrigues J. O. Guarneri A. A. Gontijo N. F.. (2012). Validation of reference genes for expression analysis in the salivary gland and the intestine of *Rhodnius prolixus* (Hemiptera, Reduviidae) under different experimental conditions by quantitative real-time PCR. BMC. Res. Notes 5:128. doi: 10.1186/1756-0500-5-128, PMID: 22395020 PMC3337225

[ref35] Pan H. Yang X. Siegfried B. D. Zhou X. (2015). A comprehensive selection of reference genes for RT-qPCR analysis in a predatory lady beetle, *Hippodamia convergens* (Coleoptera: Coccinellidae). PLoS One 10:e0125868. doi: 10.1371/journal.pone.0125868, PMID: 25915640 PMC4411045

[ref36] Pfaffl M. W. (2001). A new mathematical model for relative quantification in real-time RT–PCR. Nucleic Acids Res. 29:e45. doi: 10.1093/nar/29.9.e45, PMID: 11328886 PMC55695

[ref37] Pfaffl M. W. Tichopad A. Prgomet C. Neuvians T. P. (2004). Determination of stable housekeeping genes, differentially regulated target genes and sample integrity: BestKeeper—excel-based tool using pair-wise correlations. Biotechnol. Lett. 26, 509–515. doi: 10.1023/B:BILE.0000019559.84305.47, PMID: 15127793

[ref38] Ponton F. Chapuis M. P. Pernice M. Sword G. A. Simpson S. J. (2011). Evaluation of potential reference genes for reverse transcription-qPCR studies of physiological responses in *Drosophila melanogaster*. J. Insect Physiol. 57, 840–850. doi: 10.1016/j.jinsphys.2011.03.014, PMID: 21435341

[ref39] Radonic A. Thulke S. Mackay I. M. Landt O. Siegert W. Nitsche A. (2004). Guideline to reference gene selection for quantitative real-time PCR. Biochem. Biophys. Res. Commun. 313, 856–862. doi: 10.1016/j.bbrc.2003.11.177, PMID: 14706621

[ref40] Renard M. Vanhauwaert S. Vanhomwegen M. Rihani A. Vandamme N. Goossens S.. (2018). Expressed repetitive elements are broadly applicable reference targets for normalization of reverse transcription-qPCR data in mice. Sci. Rep. 8:7642. doi: 10.1038/s41598-018-25389-6, PMID: 29769563 PMC5955877

[ref41] Sang J. Wang Z. Li M. Cao J. Niu G. Xia L.. (2018). ICG: a wiki-driven knowledgebase of internal control genes for RT-qPCR normalization. Nucleic Acids Res. 46, D121–D126. doi: 10.1093/nar/gkx875, PMID: 29036693 PMC5753184

[ref42] Selvey S. Thompson E. W. Matthaei K. Lea R. A. Irving M. G. Griffiths L. R. (2001). Beta-actin--an unsuitable internal control for RT-PCR. Mol. Cell. Probes 15, 307–311. doi: 10.1006/mcpr.2001.0376, PMID: 11735303

[ref43] Silver N. Best S. Jiang J. Thein S. L. (2006). Selection of housekeeping genes for gene expression studies in human reticulocytes using real-time PCR. BMC Mol. Biol. 7:33. doi: 10.1186/1471-2199-7-33, PMID: 17026756 PMC1609175

[ref44] Sun M. Lu M. X. Tang X. T. Du Y. Z. (2015). Exploring valid reference genes for quantitative real-time PCR analysis in *Sesamia inferens* (Lepidoptera: Noctuidae). PLoS One 10:e0115979. doi: 10.1371/journal.pone.0115979, PMID: 25585250 PMC4293147

[ref45] Sun H. F. Meng Y. P. Cui G. M. Cao Q. F. Li J. Liang A. H. (2009). Selection of housekeeping genes for gene expression studies on the development of fruit bearing shoots in Chinese jujube (*Ziziphus jujube* mill.). Mol. Biol. Rep. 36, 2183–2190. doi: 10.1007/s11033-008-9433-y, PMID: 19109762

[ref46] Sun H. Song Y. Du J. Wang X. Cheng Z. (2016). Identification and tissue distribution of chemosensory protein and odorant binding protein genes in *Athetis dissimilis* (Lepidoptera: Noctuidae). Appl. Entomol. Zool. 51, 409–420. doi: 10.1007/s13355-016-0413-8

[ref47] Vandesompele J. De Preter K. Pattyn F. Poppe B. Van Roy N. De Paepe A.. (2002). Accurate normalization of real-time quantitative RT-PCR data by geometric averaging of multiple internal control genes. Genome Biol. 3:research0034. doi: 10.1186/gb-2002-3-7-research0034, PMID: 12184808 PMC126239

[ref48] Wan Q. Chen S. Shan Z. Yang Z. Chen L. Zhang C.. (2017). Stability evaluation of reference genes for gene expression analysis by RT-qPCR in soybean under different conditions. PLoS One 12:e0189405. doi: 10.1371/journal.pone.0189405, PMID: 29236756 PMC5728501

[ref49] Xie J. Liu T. Khashaveh A. Yi C. Liu X. Zhang Y. (2021). Identification and evaluation of suitable reference genes for RT-qPCR analysis in *Hippodamia variegata* (Coleoptera: Coccinellidae) under different biotic and abiotic conditions. Front. Physiol. 12:669510. doi: 10.3389/fphys.2021.669510, PMID: 34079474 PMC8165390

[ref50] Xie F. Xiao P. Chen D. Xu L. Zhang B. (2012). miRDeepFinder: a miRNA analysis tool for deep sequencing of plant small RNAs. Plant Mol. Biol. 80, 75–84. doi: 10.1007/s11103-012-9885-2, PMID: 22290409

[ref51] Yan Z. Gao J. Lv X. Yang W. Wen S. Tong H.. (2016). Quantitative evaluation and selection of reference genes for quantitative RT-PCR in mouse acute pancreatitis. Biomed. Res. Int. 2016:8367063. doi: 10.1155/2016/8367063, PMID: 27069927 PMC4812220

[ref52] Yang Q. Li Z. Cao J. Zhang S. Zhang H. Wu X.. (2014). Selection and assessment of reference genes for quantitative PCR normalization in migratory locust *Locusta migratoria* (Orthoptera: Acrididae). PLoS One 9:e98164. doi: 10.1371/journal.pone.0098164, PMID: 24887329 PMC4041718

[ref53] Yin J. Sun L. Zhang Q. Cao C. (2020). Screening and evaluation of the stability of expression of reference genes in *Lymantria dispar* (Lepidoptera: Erebidae) using qRT-PCR. Gene 749:144712. doi: 10.1016/j.gene.2020.144712, PMID: 32360412

[ref54] Zhang S. An S. Li Z. Wu F. Yang Q. Liu Y.. (2015). Identification and validation of reference genes for normalization of gene expression analysis using qRT-PCR in *Helicoverpa armigera* (Lepidoptera: Noctuidae). Gene 555, 393–402. doi: 10.1016/j.gene.2014.11.038, PMID: 25447918

[ref55] Zhao X. Fu J. Jiang L. Zhang W. Shao Y. Jin C.. (2018). Transcriptome-based identification of the optimal reference genes as internal controls for quantitative RT-PCR in razor clam (*Sinonovacula constricta*). Genes Genomics 40, 603–613. doi: 10.1007/s13258-018-0661-9, PMID: 29892942

[ref56] Zheng Y. T. Li H. B. Lu M. X. Du Y. Z. (2014). Evaluation and validation of reference genes for qRT-PCR normalization in *Frankliniella occidentalis* (Thysanoptera: Thripidae). PLoS One 9:e111369. doi: 10.1371/journal.pone.0111369, PMID: 25356721 PMC4214748

