# Supplementary material for: Selection and Validation of Reference Genes for Quantitative Real-Time PCR Normalization in Athetis dissimilis (Lepidoptera: Noctuidae) Under Different Conditions
Source: Front Physiol. 2022 Feb 22;13:842195. doi: 10.3389/fphys.2022.842195 (PMC8902415; doi:10.3389/fphys.2022.842195)
Supplement: Supplementary file 1 [file Data_Sheet_1.docx]

**Supplementary:**

Table S1 Primers for clone

| Gene Name | Primer Sequence (5’–3’) Forward/Reverse |
| --- | --- |
| EF1-α | TACTATGTCACCATCATCGAC |
|  | GTGTATCCGTTTGAGATTTGACC |
| 18S | ATTAAGCCATGCATGTCTCAG |
|  | AAGTGTACTCATTCCGATTACG |
| 28S | GGGTAAACCTGCGAAACTCG |
|  | TTTCAAGACGGGTCCTGCGAG |
| β-TUB | AACATGGTGCCGTTCCCACG |
|  | CACCAGGTCGTTCATGTTGC |
| α-TUB | GACTCCTTCAACACCTTCTTCAGC |
|  | CCAAGTTGGTCTGGAACTCGGT |
| RPL40 | CCCTCACGGGTAAAACCATCACC |
|  | CAGGTTGTTGGTGTGTCCACACTTGG |
| RPL32 | GTATACAGGCCGACAATCGTC |
|  | GAGACGCCGTGAGCGATCTC |
| AK | GACCCCATCATCGAGGACTACC |
|  | CGTACTCGGTGAGACCCATGCG |
| β-ACT | GAGAAGATCTGGCACCACACCT |
|  | GTTGGCGTACAGGTCCTTACG |

Table S2 Sensitivities of A. dissimilis to two insecticides

| Insecticides | Slope±SE | LC50 (95% confidence limit)/(mg/L) | Relative toxicity index |
| --- | --- | --- | --- |
| Chlorantraniliprole | 1.46±0.27 | 1.544（0.8546-2.310） | 5.94 |
| Lambda-cyhalothrin | 1.10±0.34 | 4.878（2.323-14.23） | 3.27 |

Table S3. Detailed distribution of cycle threshold (Ct) value information

| Experimental conditions | Statistics | Reference gene | | | | | | | | | |
| --- | --- | --- | --- | --- | --- | --- | --- | --- | --- | --- | --- |
|  |  | *18S* | *28S* | *AK* | *α-TUB* | *β-ACT* | *β-TUB* | *EF-1α* | *GAPDH* | *RPL32* | *RPL40* |
| Developmental stages | Group | 10 | 10 | 10 | 10 | 10 | 10 | 10 | 10 | 10 | 10 |
|  | Mean | 7.04 | 14.83 | 19.84 | 18.40 | 17.91 | 19.65 | 17.44 | 18.70 | 19.21 | 18.34 |
|  | Min Ct | 6.03 | 13.79 | 17.72 | 17.10 | 15.04 | 18.16 | 16.33 | 16.44 | 18.32 | 17.66 |
|  | Max Ct | 7.69 | 15.73 | 22.85 | 19.48 | 21.66 | 21.34 | 18.28 | 21.07 | 20.49 | 18.71 |
| Larva tissues | Group | 7 | 7 | 7 | 7 | 7 | 7 | 7 | 7 | 7 | 7 |
|  | Mean | 6.53 | 14.39 | 20.70 | 17.91 | 19.21 | 19.40 | 17.11 | 19.15 | 19.95 | 19.20 |
|  | Min Ct | 6.12 | 13.72 | 17.65 | 17.09 | 14.57 | 17.81 | 16.53 | 18.35 | 19.66 | 18.77 |
|  | Max Ct | 7.29 | 16.06 | 24.12 | 18.42 | 21.65 | 20.35 | 18.11 | 20.42 | 20.87 | 19.85 |
| Adult tissues | Group | 6 | 6 | 6 | 6 | 6 | 6 | 6 | 6 | 6 | 6 |
|  | Mean | 7.25 | 15.22 | 18.75 | 19.55 | 18.26 | 20.39 | 18.36 | 18.50 | 20.15 | 20.34 |
|  | Min Ct | 6.28 | 14.27 | 15.80 | 18.56 | 15.46 | 18.65 | 16.95 | 16.61 | 18.65 | 18.09 |
|  | Max Ct | 10.76 | 17.92 | 22.53 | 22.57 | 20.87 | 23.84 | 21.63 | 21.54 | 24.45 | 24.57 |
| Diets | Group | 4 | 4 | 4 | 4 | 4 | 4 | 4 | 4 | 4 | 4 |
|  | Mean | 6.21 | 14.46 | 19.26 | 18.11 | 15.45 | 19.23 | 16.38 | 18.77 | 19.25 | 18.56 |
|  | Min Ct | 6.12 | 14.30 | 19.06 | 17.86 | 15.12 | 18.97 | 16.14 | 17.95 | 19.11 | 18.40 |
|  | Max Ct | 6.30 | 14.57 | 19.45 | 18.37 | 15.75 | 19.39 | 16.55 | 19.59 | 19.36 | 18.69 |
| Insecticide-induced stress | Group | 8 | 8 | 8 | 8 | 8 | 8 | 8 | 8 | 8 | 8 |
|  | Mean | 6.38 | 14.20 | 18.68 | 17.99 | 15.95 | 19.97 | 16.78 | 19.47 | 18.06 | 18.16 |
|  | Min Ct | 5.91 | 13.94 | 17.37 | 17.30 | 14.89 | 19.18 | 15.71 | 18.72 | 17.68 | 17.78 |
|  | Max Ct | 6.72 | 14.60 | 20.95 | 18.80 | 16.90 | 20.84 | 17.76 | 20.70 | 18.46 | 18.56 |
| Temperature treatment | Group | 9 | 9 | 9 | 9 | 9 | 9 | 9 | 9 | 9 | 9 |
|  | Mean | 6.48 | 14.38 | 19.45 | 17.94 | 16.68 | 19.50 | 16.65 | 19.36 | 19.39 | 18.58 |
|  | Min Ct | 6.16 | 13.97 | 18.51 | 17.57 | 15.71 | 19.11 | 16.23 | 18.63 | 18.66 | 18.23 |
|  | Max Ct | 7.06 | 14.70 | 20.37 | 18.34 | 17.60 | 19.82 | 17.16 | 20.58 | 19.96 | 18.79 |
| Starvation treatment | Group | 2 | 2 | 2 | 2 | 2 | 2 | 2 | 2 | 2 | 2 |
|  | Mean | 6.76 | 15.07 | 18.42 | 18.94 | 17.02 | 20.45 | 18.40 | 19.72 | 18.50 | 18.53 |
|  | Min Ct | 6.68 | 14.90 | 18.34 | 18.90 | 17.01 | 20.23 | 18.30 | 19.70 | 17.86 | 18.19 |
|  | Max Ct | 6.83 | 15.23 | 18.50 | 18.98 | 17.02 | 20.68 | 18.50 | 19.74 | 19.14 | 18.86 |

‘Group’ means how many states or tissues the genes are studied in.


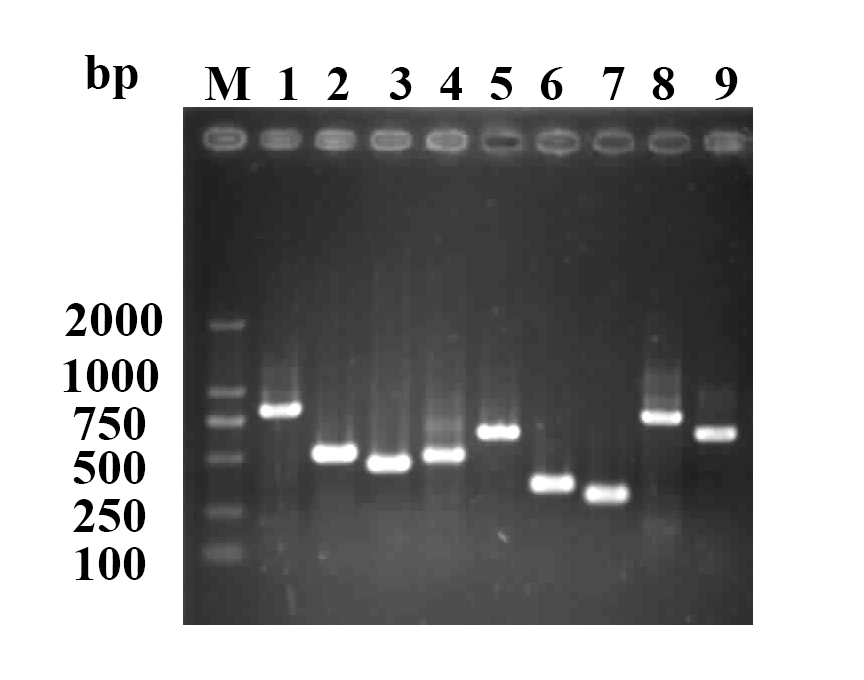


Figure S1. PCR amplification products of the 9 cloned candidate reference genes. M: DNA molecular weight marker, 1: EF1-α, 2: 18S, 3: 28S, 4: β-TUB, 5: α-TUB, 6: RPL40, 7: RPL32, 8: AK, 9: β-ACT.


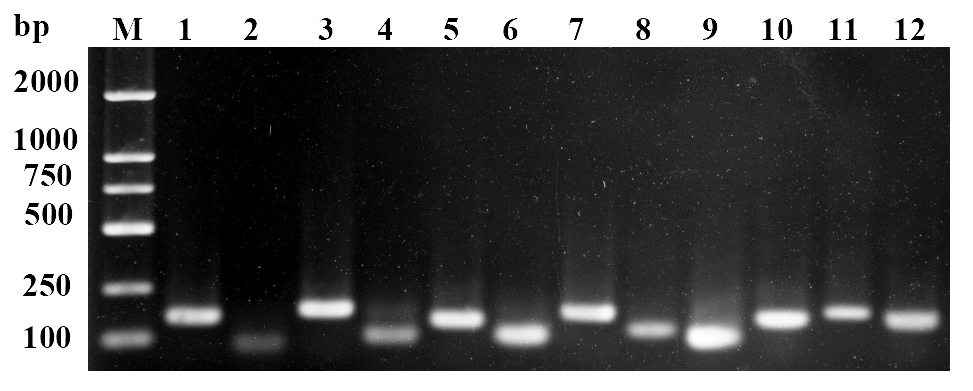


Figure S2. The amplicon length of the 10 candidate reference genes and 2 target genes. M: DNA molecular weight marker, 1: GAPDH, 2: β-ACT, 3: RPL32, 4: RPL40, 5: EF1-α, 6: α-TUB, 7: β-TUB, 8: 18S, 9: 28S, 10: AK, 11: CSP1, 12: SOD.


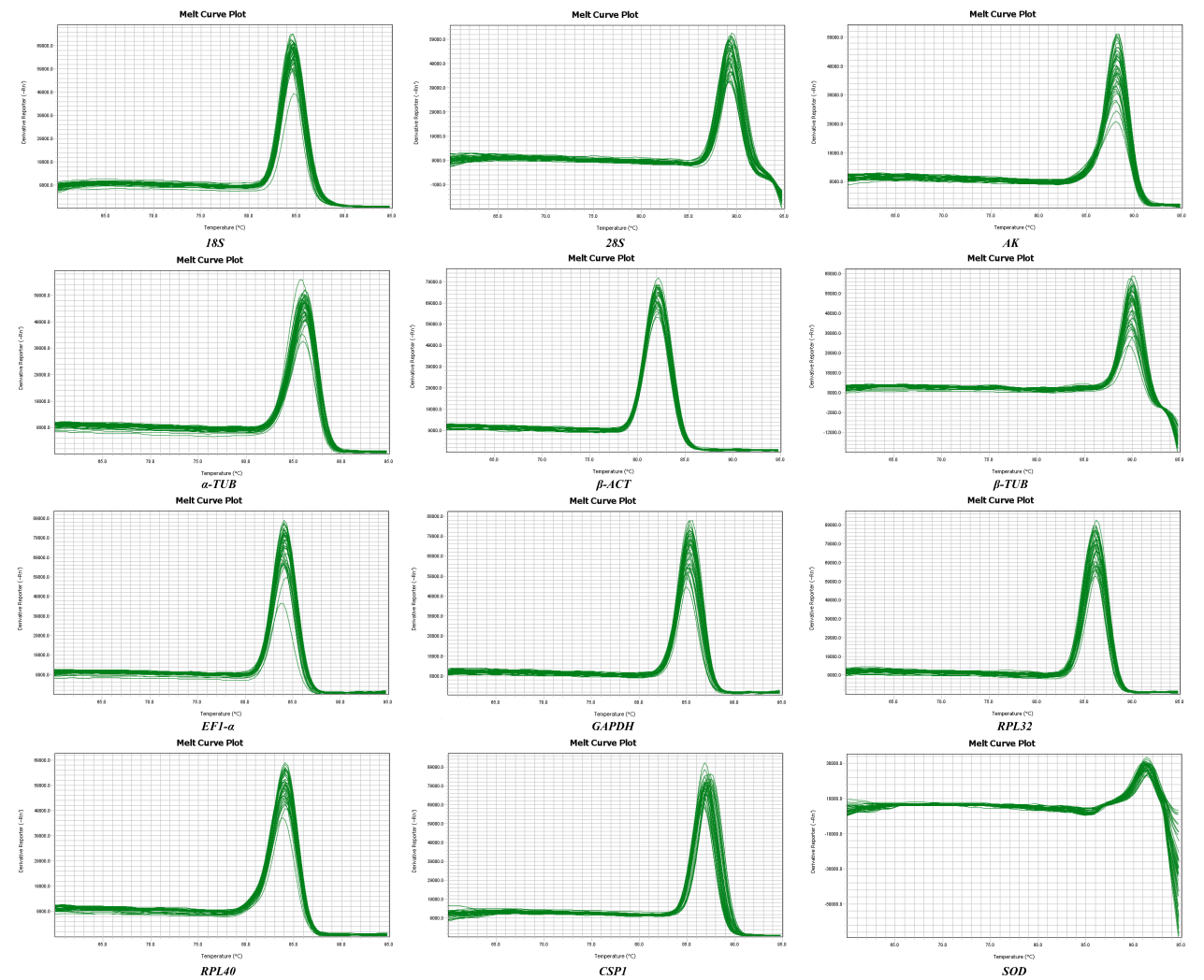


Figure S3. Melting curves for the selected genes.
